# Data on horizontal and vertical movements of zebrafish during appetitive conditioning

**DOI:** 10.1016/j.dib.2016.10.007

**Published:** 2016-10-22

**Authors:** Neil Merovitch, Jillian M. Doyle, Russell C. Wyeth, Matthew R. Stoyek, Michael K. Schmidt, Florentin M. Wilfart, Alan Fine, Roger P. Croll

**Affiliations:** a Department of Physiology & Biophysics, Dalhousie University, Halifax, NS B3H 4R2, Canada; b Department of Biology, St. Francis Xavier University, Antigonish, NS B2G 2W5, Canada; c Department of Medical Neuroscience, Dalhousie University, Halifax, NS B3H 4R2, Canada; d Department of Anesthesia, Pain Management and Perioperative Medicine, Dalhousie University, Halifax, NS B3H 4R2, Canada; e School of Biomedical Engineering, Dalhousie University, Halifax, NS B3H 4R2, Canada

**Keywords:** Zebrafish, Learning, Memory, Classical conditioning, High-throughput, Behaviour

## Abstract

This article provides supporting data for the research article “A simple automated system for appetitive conditioning of zebrafish in their home tanks” (J.M. Doyle, N. Merovitch, R.C. Wyeth, M.R. Stoyek, M. Schmidt, F. Wilfart, A. Fine, R.P. Croll, 2016) [1]. In that article, we described overall movements of zebrafish toward a food source as a response to auditory or visual cues as conditioned stimuli in a novel learning paradigm. Here, we describe separate analyses of the vertical and horizontal components of the learned response. These data provide evidence that the conditioning might result from both classical conditioning of an innate response of zebrafish to move to the surface in response to food cues and secondary conditioning of the fish to associate a food presentation with a specific location in the tank. Movement data from the twenty trial acquisition period and probe trials from 2–32 days post conditioning are included.

**Specifications Table**

**Table:** 

Subject area	*Biology*
More specific subject area	*Behavioural neuroscience*
Type of data	*Graphs*
How data were acquired	*Logitech Webcam C930s & Honeywell HCM5748 cameras*
Data format	*Filtered, analyzed*
Experimental factors	*Condition: paired vs specifically unpaired presentation of a stimulus with food reward. Acquisition: trials 1–20. Retention time: 2–32 days.*
Experimental features	*Tracking of zebrafish movement during an appetitive learning paradigm which uses auditory or visual cues as conditioned stimuli*
Data source location	*Department of Physiology & Biophysics, Dalhousie University, Halifax, Nova Scotia, Canada*
Data accessibility	*Data are supplied with this article*

**Value of the data**

• Contributes data to compare to those obtained from other zebrafish conditioning paradigms.
• Establishes a behavioural baseline for zebrafish movement in a home tank environment.
• Provides data for comparison in zebrafish studies where learning behaviour or memory retention is affected.

## 1 Data

**Fig. 1:**
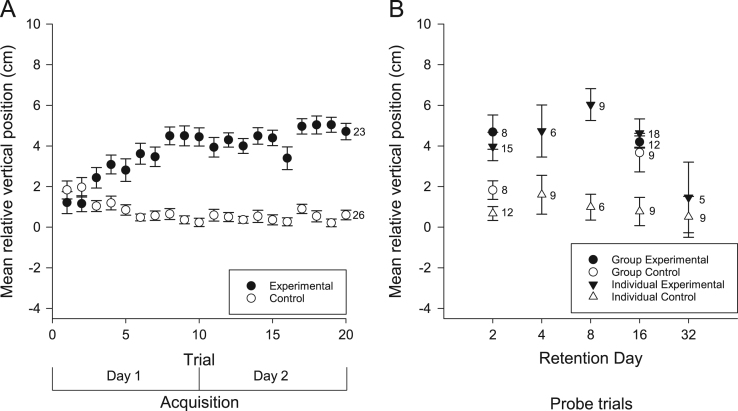
Vertical movements of adult zebrafish during acquisition and retention of an auditory appetitive paradigm. (A) Zebrafish in the experimental group moved vertically from their initial positions towards the food source as a result of conditioning to the auditory stimulus. This response increased throughout the training trials. Zebrafish in the control group did not move to the surface in response to the auditory stimulus. (B) When tested for retention on various days, both trained groups and trained individuals moved closer to the surface compared to controls. Data points are mean vertical position before the FM tone sweep minus mean vertical position during the FM tone sweep. Numbers beside data points represent replicates for individuals (single fish) or groups (each containing 5 fish) in each condition. Error bars=±S.E.M.

**Fig. 2:**
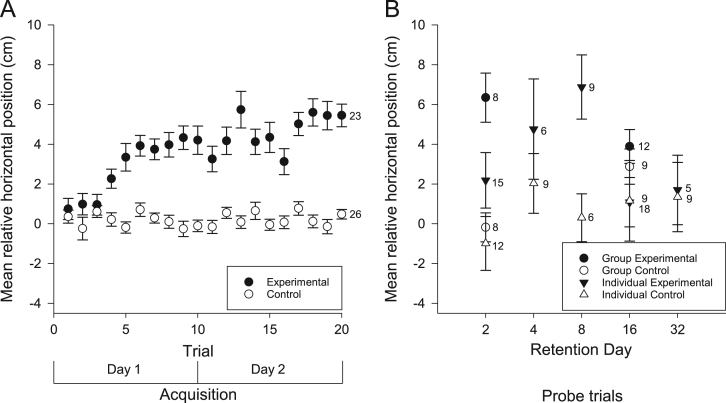
Horizontal movements of adult zebrafish during acquisition and retention of an auditory appetitive paradigm. (A) Adult zebrafish in the experimental group moved laterally from their initial positions towards the food source as a result of conditioning to the auditory stimulus. This response increased throughout the training trials. Zebrafish in the control group did not move laterally towards the food source in response to the auditory stimulus. (B) When tested for retention on various days, trained groups and trained individuals moved, laterally, towards the food source in comparison to the controls. Data points are mean horizontal position before the FM tone sweep minus mean horizontal position during the FM tone sweep. Numbers beside data points represent replicates for individuals (single fish) or groups (each containing 5 fish) in each condition. Error bars=± S.E.M.

**Fig. 3:**
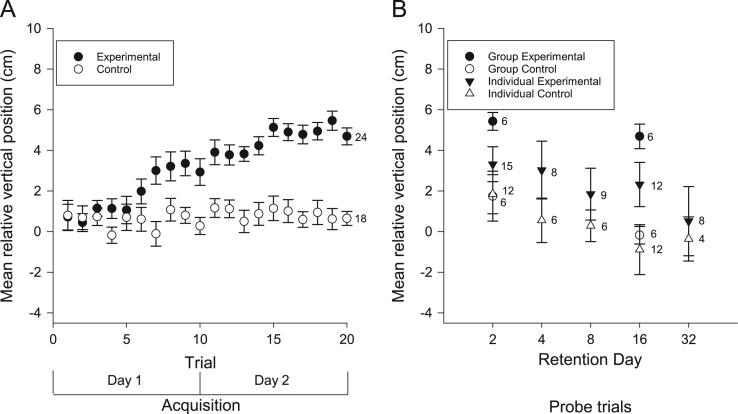
Vertical movements of adult zebrafish during acquisition and retention of a visual appetitive paradigm. (A) Adult zebrafish in the experimental group moved vertically from their initial positions towards the surface as a result of conditioning to the visual stimulus. This response increased throughout the training trials. Zebrafish in the control group did not move vertically towards the food source in response to the visual stimulus. (B) When the fish were tested for retention on various days, both trained groups and individuals moved more towards the surface compared to controls. Data points are mean vertical position before the LED illumination minus mean vertical position during LED illumination. Numbers beside data points represent replicates for individuals (single fish) or groups (each containing 5 fish) in each condition. Error bars=± S.E.M.

**Fig. 4:**
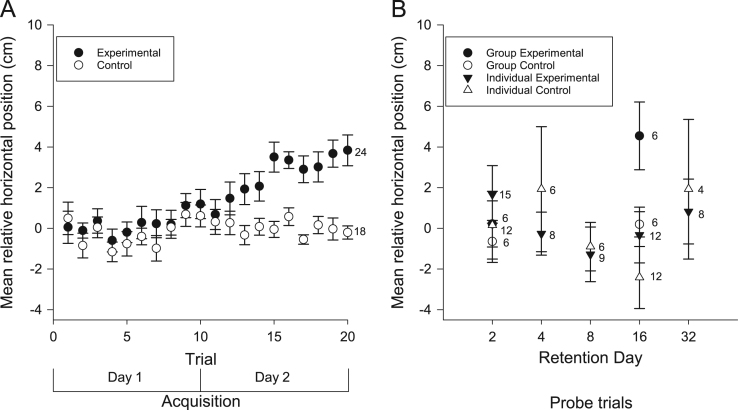
Horizontal movements of adult zebrafish during acquisition and retention of a visual appetitive paradigm. (A) Adult zebrafish in the experimental group moved laterally from their initial positions towards the food source as a result of conditioning to the visual stimulus. This response increased throughout the training trials. Zebrafish in the control group did not move laterally towards the food source in response to the visual stimulus. (B) When tested for retention on various, trained groups moved closer, laterally, towards the food source compared to controls. The individual fish did not move closer to the food source when compared with the controls. Data points are mean horizontal position before the LED illumination sweep minus mean horizontal position during the LED illumination. Numbers beside data points represent replicates for individuals (single fish) or groups (each containing 5 fish) in each condition. Error bars=± S.E.M.

**Fig. 5:**
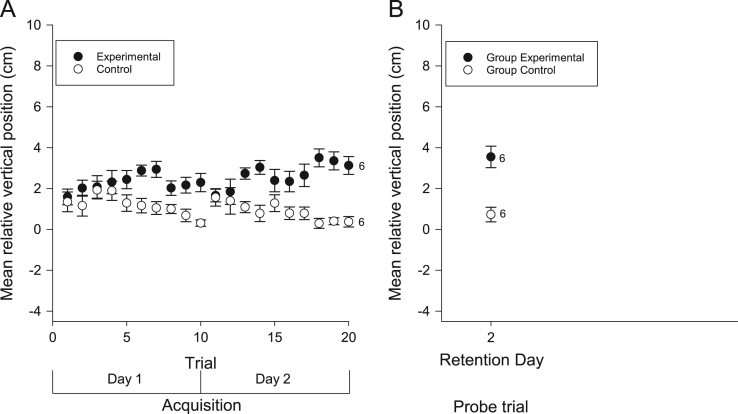
Vertical movements of juvenile zebrafish during acquisition and retention of an auditory appetitive paradigm. (A) Zebrafish (49 dpf) in the experimental group moved towards the surface from their initial positions towards the surface as a result of conditioning to the auditory stimulus. This response increased throughout the training trials. Zebrafish in the control group did not move vertically toward the food source in response to the auditory stimulus. (B) When tested for retention in groups after 2 days, trained fish moved closer to the surface compared to controls. Data points are mean vertical position before the FM tone sweep minus mean vertical position during the FM tone sweep. Numbers beside data points represent replicates for groups of 5 fish in each condition. Error bars=± S.E.M.

**Fig. 6:**
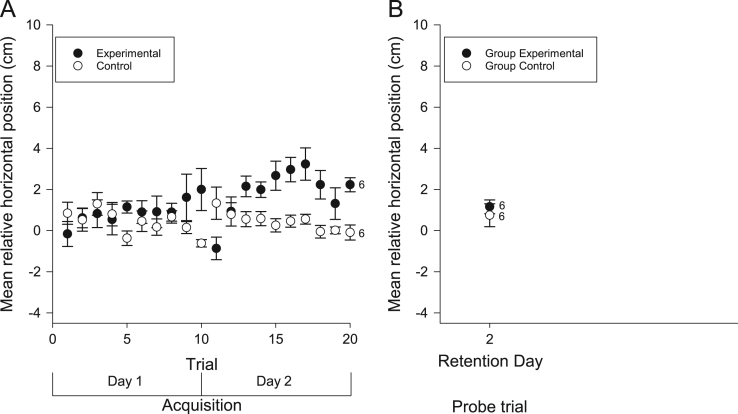
Horizontal movements of juvenile zebrafish during acquisition and retention of an auditory appetitive paradigm. (A) Zebrafish (49 dpf) in the experimental group moved laterally from their initial positions towards the surface as a result of conditioning to the auditory stimulus. This response increased throughout the training trials. Zebrafish in the control group did not move laterally toward the food source in response to the auditory stimulus. (B) When tested for retention in groups after 2 days, trained fish did not move closer, laterally, to the food source when compared to controls. Data points are mean horizontal position before the FM tone sweep minus mean horizontal position during the FM tone sweep. Numbers beside data points represent replicates for groups of 5 fish in each condition. Error bars=± S.E.M.

We examined the vertical and horizontal components of zebrafish movement in response to a sound (Figs. 1 and 2A) or light (Figs. 3 and 4A) paired with food over twenty trials. We also examined the vertical and horizontal movement of juvenile zebrafish (49 days after fertilization) in response to a sound paired with food (Figs. 5 and 6A) over twenty trials.

In addition to the data collected during acquisition of conditioning, we also examined the vertical and horizontal movements of the zebrafish in probe trials when the conditioned stimuli were presented alone some days after training. Retention was examined separately in both individual and groups of adult zebrafish presented with the auditory (Figs. 1 and 2B) and visual (Figs. 3 and 4B) conditioned stimuli and in groups of juvenile zebrafish presented only with the auditory stimulus (Figs. 5 and 6B).

## 2 Experimental design, materials and methods

### 2.1 Acquisition period

Training consisted of 10 pairings of the auditory or visual stimulus with food on each of two consecutive days. Conditioning was performed by either playing FM tone sweeps or illuminating green LEDs for a 20-second period. The conditioned stimulus was immediately followed by the presentation of the food reward. In trials with control fish, the unconditioned stimulus (food) did not immediately follow the conditioned stimulus, but was instead administered at variable times after the conditioned stimulus.

### 2.2 Probe trials

Probe trials to test memory retention were conducted at various times after training. Fish were either tested in the groups in which they were trained or tested individually. For testing single fish, one animal at a time was removed from the group tank and transferred to a new tank one day before testing. On the day of testing, fish were exposed to the stimulus to which they were conditioned for 20 s without the food reward to test the memory of the association. Each group or individual fish was given only a single probe trial at 2, 4, 8, 16 or 32 days after training.

### 2.3 Data collection and analysis

Trials were recorded for the 20 s immediately before exposure to the auditory or visual conditioned stimulus and the 20 s period during presentation of the conditioned stimulus. Videograms [2] were created in Matlab to track the average position of the group of fish. The average vertical and horizontal positions of the fish in each tank were calculated for the 20 s before the presentation of the conditioned stimulus and subtracted from the average coordinates during presentation of the stimulus. Positive vertical scores correspond to upward movements towards the surface, and positive horizontal scores correspond to a lateral movement toward the end of the tank with the food source, regardless of initial positions.

## 3 Statistical analysis

Linear mixed-effects models were used to analyse the acquisition data. Two-way full factorial analyses of variance (ANOVAs; with conditioning and probe time factors) and Welch two sample *t*-tests were conducted for the probe trials in adults and juveniles, respectively (For full statistical analysis see Appendices B–D in [1]).
